# Lateral Arm Flap: Its Usage as Pedicle and Free Flap

**DOI:** 10.7759/cureus.12136

**Published:** 2020-12-18

**Authors:** Sami Ullah, Muhammad Asif, Muhammad Ubaid, Amna Khalid, Majid Khan, Mohammad Fazlur Rahman

**Affiliations:** 1 Plastic and Reconstructive Surgery, Shaukat Khanum Memorial Cancer Hospital and Research Centre, Lahore, PAK; 2 Plastic Surgery, Cancer Foundation Hospital, Karachi, PAK; 3 Community Health Sciences, Aga Khan University, Karachi, PAK; 4 Plastic and Reconstructive Surgery, Aga Khan University Hospital, Karachi, PAK

**Keywords:** free lateral arm flap (flaf), pedicle lateral arm flap (plaf), extended lateral arm flap (elaf)

## Abstract

Introduction

The lateral arm flap is an excellent flap for both local and microvascular reconstruction. For local reconstruction on the upper extremity or as a distant microvascular flap, its advantages include short operation time, thin pliable tissue, non-dominant vessel, and minimal donor site morbidity. Moreover, it fulfills the goal of optimal reconstruction of form, function, and aesthetics. The objective is to share our experience of using the lateral arm flap, both as a free flap and as a pedicled flap.

Methods

After taking exemption from the ethical review committee (ERC) of Aga Khan University Hospital, a retrospective data analysis of patients who had undergone lateral arm flap at the Plastic and Reconstructive Surgery department of the Aga Khan University Hospital was carried out from January 2012 to December 2019. The data examined included the patient's age, gender, diagnosis, location of the defect, size of the flap, and outcome of the flap at three weeks post-operation. For free flaps, data of the recipient artery used for anastomosis and the number of veins anastomosed were also included.

Results

Over a period of eight years, 33 lateral arm flaps were performed, including 23 free flaps and 10 pedicled flaps. The average size of the free flap was 12x6 cm and that of the pedicled flap was 8x5 cm. In the free-flap group, there was a failure in three flaps, two of which were due to arterial anastomosis in the zone of injury. There were no failures in the pedicled flap group.

Conclusion

The lateral arm flap is a reliable flap, with consistent anatomy, which can be used for coverage in different parts of the body.

## Introduction

One aspect of reconstructive surgery is to replace any lost tissue with similar tissue. With recent advancements in the field of microsurgery, it is now possible to achieve better aesthetic results with reduced donor site morbidity.

For soft tissue replacement, radial forearm flap has been the procedure of choice ever since its description appeared in the Chinese literature. However, the radial forearm flap had drawbacks such as donor site morbidity and the loss of a major artery of the forearm. This was replaced by the anterolateral thigh flap, which was described by Song et al. in 1984 and popularized by Fu Chan Wei. However, its drawback was that it was a bulky flap [1-2].

A lateral arm free flap was first described by Katsaros et al. as a versatile free flap that could be used in many reconstructions [3]. It is now commonly used in upper extremity reconstruction, both as a reverse pedicle and as a free flap. It is well-suited for covering defects where a thin flap is required, especially the dorsum of the hand [3-5]. This flap not only has the advantage of being thinner than the anterolateral thigh flap, but it also does not sacrifice a major artery and has acceptable donor site morbidity. However, its drawback includes a shorter pedicle and a smaller skin paddle [6]. Here, we share our experience of using the lateral arm flap both as a free flap and as a pedicled flap.

## Materials and methods

An exemption was taken from the ethical review committee (ERC) of the hospital. A retrospective review of 33 patients’ data, who underwent lateral arm flap reconstruction for various defects in the body was carried out from January 2012 to December 2019 at the Plastic and Reconstructive Surgery department of the Aga Khan University Hospital. We grouped the patients into two main categories. Only patients with three weeks of follow-up were included. Group 1 included patients who had reconstruction using a free lateral arm flap (FLAF) while Group 2 included those who had reconstruction using a pedicled lateral arm flap (PLAF). Data were collected using a standard proforma containing details about the patient’s age, sex, etiology of the defect, location of the defect, size of the flap, and outcome (survival/failure) of the flap. For free flaps, data about the recipient vessels used for anastomosis was also included. The work has been reported in line with the PROCESS (Preferred Reporting of Case Series in Surgery) criteria [7].

Operative procedure

**Figure 1:**
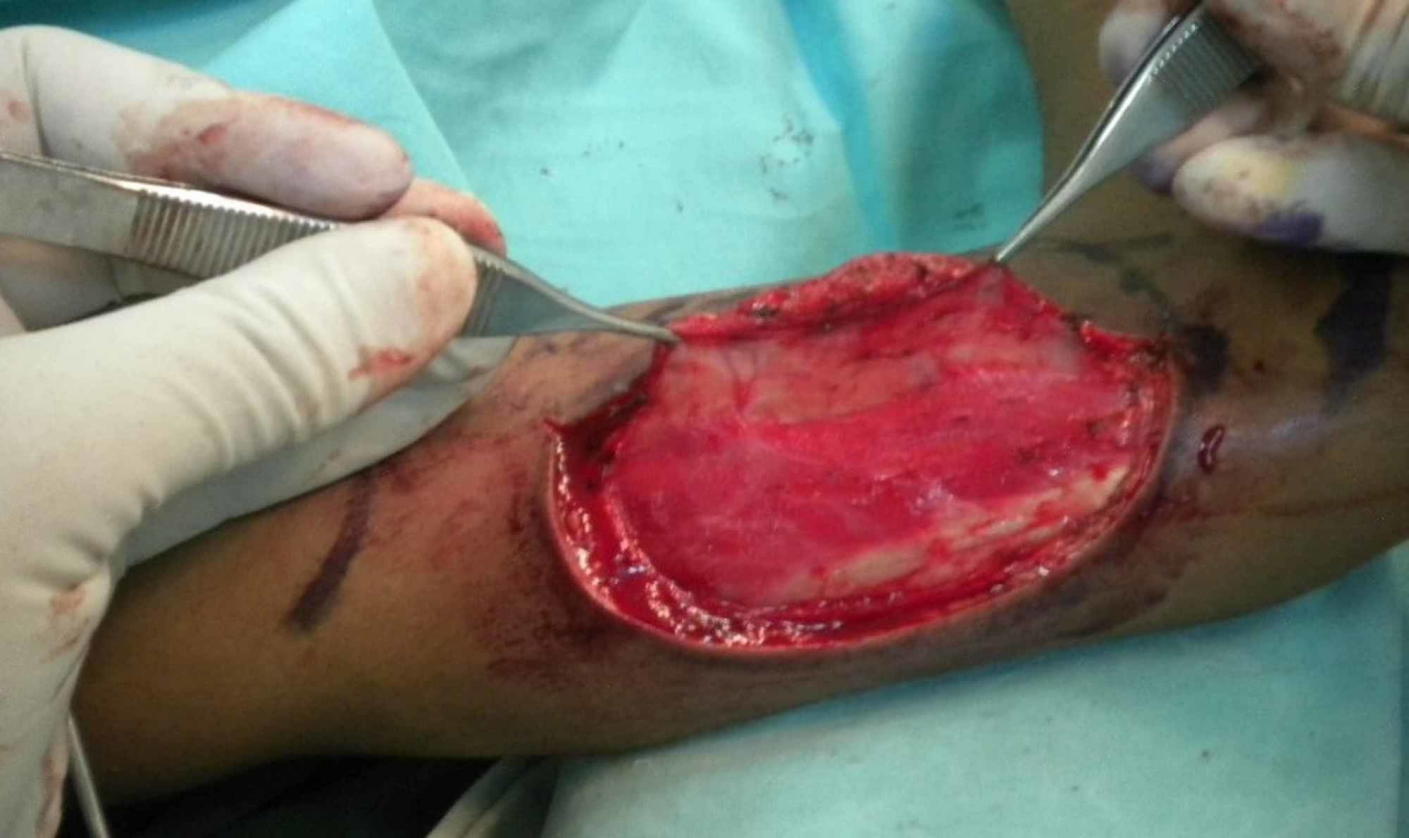
Posterior incision made first, skin and fascia elevated over the triceps muscle, identifying perforators going into the skin

**Figure 2:**
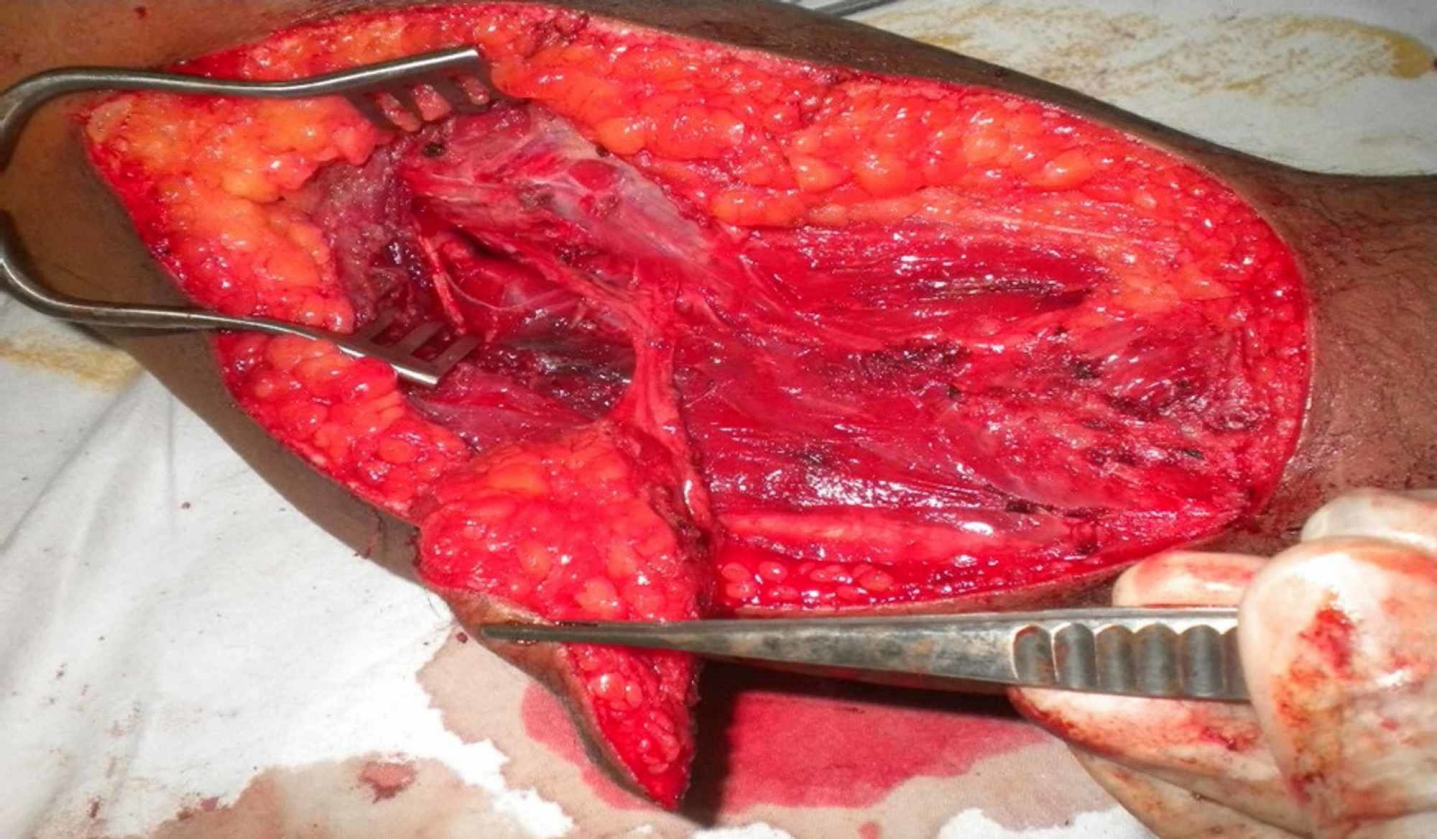
Partially elevated flap, identifying and dissecting posterior lateral collateral artery in the lateral intermuscular septum

**Figure 3:**
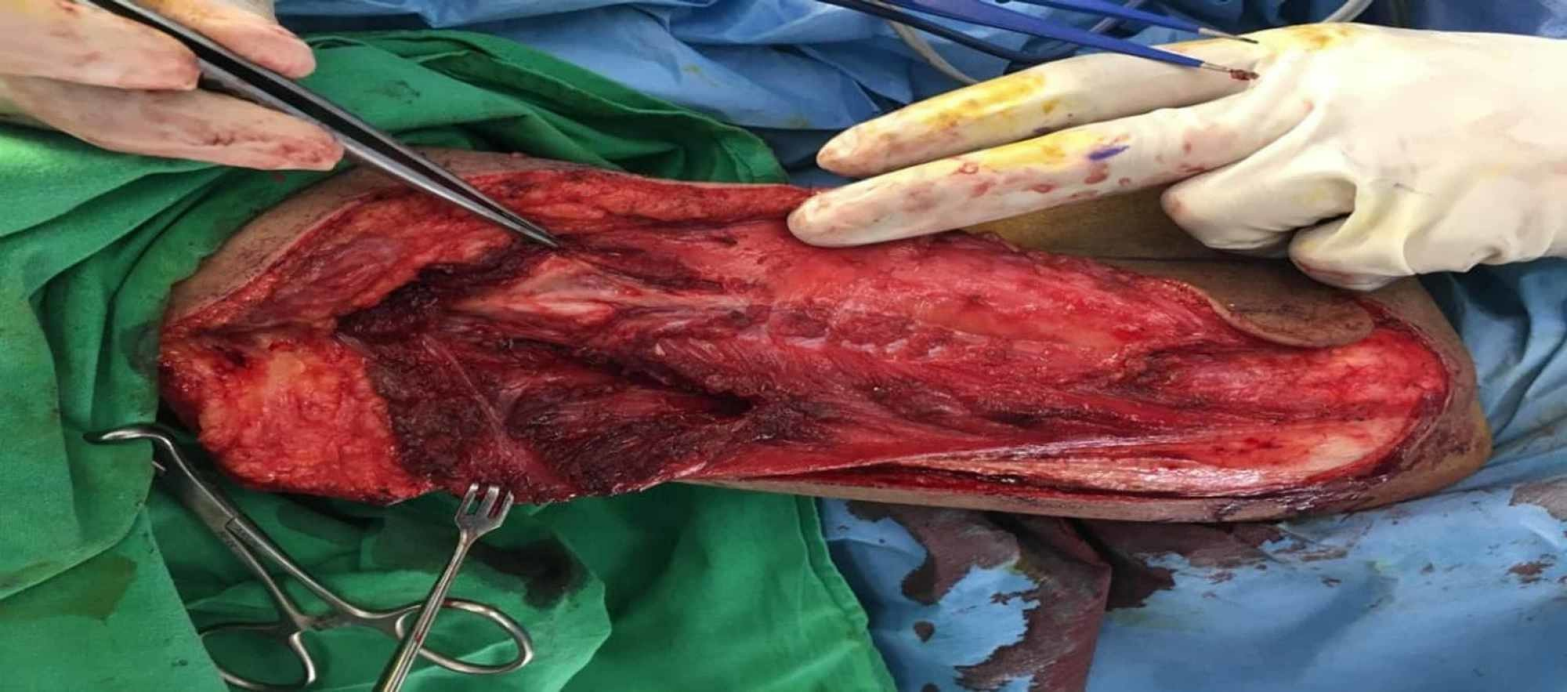
Radial nerve being identified; forceps pointing to it

**Figure 4:**
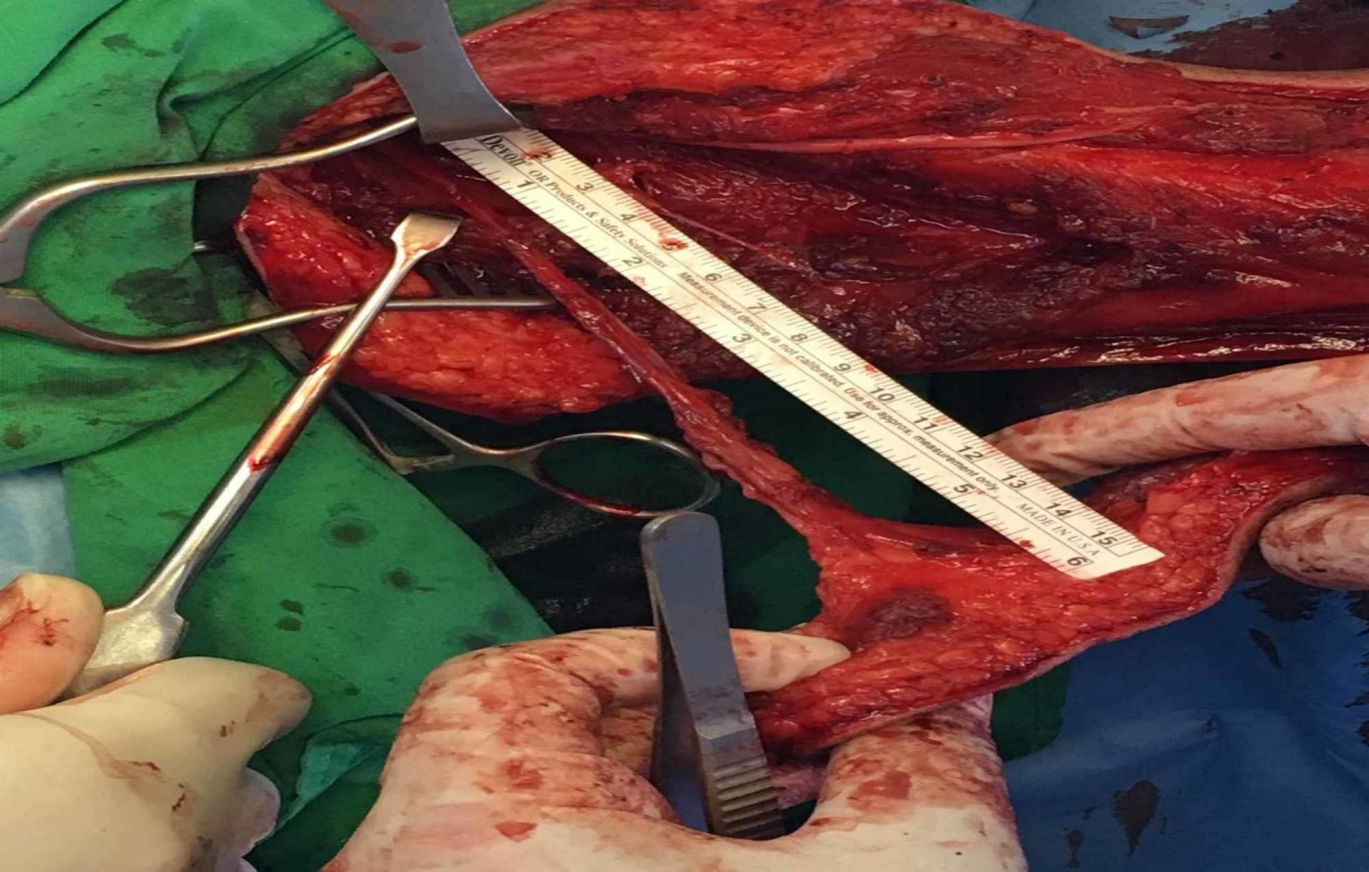
Complete elevation of flap, achieving good length of the pedicle

The operative procedure was the same as described in the literature [8]. The vascular supply of the lateral arm flap is the posterior radial collateral artery (PRCA), which is a branch of the radial collateral artery. The PRCA runs in the lateral inter-muscular septum of the arm. A line drawn from the deltoid insertion to the lateral epicondyle denotes the lateral inter-muscular septum. Anteriorly, the intermuscular septum is bounded by the brachialis and the brachioradialis and posteriorly, by the triceps. If an extended lateral arm flap (ELAF) is required then a line is drawn from the lateral epicondyle to the radial styloid. The posterior incision is made first. Skin and fascia are incised and triceps muscle identified. At this point, perforators entering into the skin are identified (Figure 1) The skin and fascia are elevated over the triceps muscle in an anterior direction and the intermuscular septum between the triceps and anterior muscles is identified. The posterior radial collateral artery is identified within the septum. An incision is made superiorly and the vessel traced and isolated (Figure 2). Once the deltoid region is reached, the main radial collateral artery is identified. At this point, the radial nerve is identified, protected, and the anterior collateral artery ligated (Figure 3). Once a good length of the vessel is obtained, the anterior incision is made. The entire flap is then elevated with the vessel and left to bleed for 20 minutes before detaching it (Figure 4).

Reverse flap

The vascular supply of this flap is based on the epicondylar and the olecranon plexi, which are supplied by the interosseous recurrent artery (IRA) and the radial recurrent artery (RRA). Harvest of the reverse pedicled flap is the same as the free flap except that the distal anastomosis is kept intact and the vessel is divided proximally.

**Figure 5:**
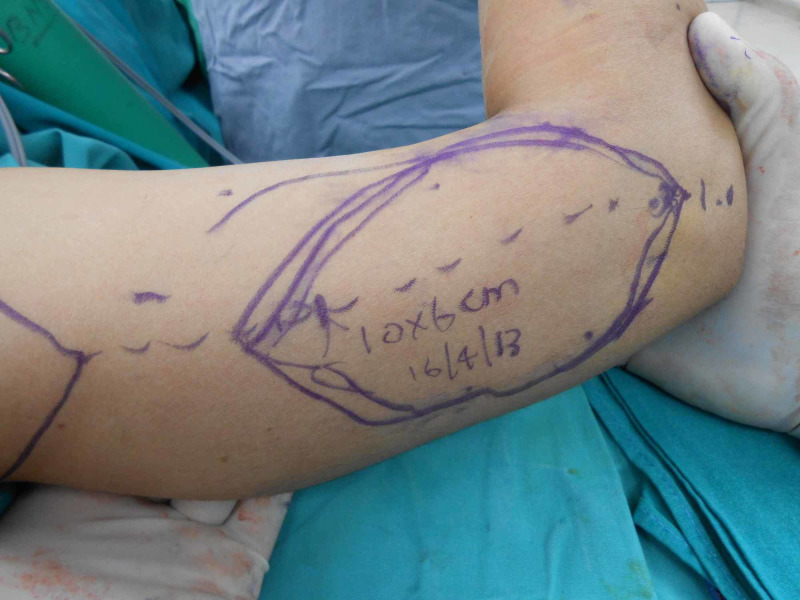
Marking of the flap

Figure 5 shows how the flap is marked.

## Results

Over the eight-year period, 33 patients underwent reconstruction using a lateral arm flap. Of these, 23 patients underwent FLAF reconstruction and 10 patients reverse PLAF (Tables 1-2).

**Table 1:** Clinical characteristics, surgical details, and outcomes of patients who underwent free lateral flap (FLAP) reconstruction

S.no	Age in Years	Gender	Aetiology	Location of Defect	Flap Size	Arterial Anastomosis	Venous Anastomosis	Outcome
1	32	Male	Trauma	Dorsum of Hand	11x6 cms	Radial Artery	Radial Vein Cephalic Vein	Survived
2	34	Male	Trauma	Dorsum of Hand	12x6 cms	Radial Artery	Radial Vein	Survived
3	14	Male	Trauma	Dorsum of Hand	7x6 cms	Radial Artery	Radial Vein	Failed
4	34	Male	Post Burn Contracture	Dorsum of Hand	15x6 cms	Radial Artery	Radial Vein	Survived
5	23	Male	Trauma	Dorsum of Foot	12x7 cms	Anterior Tibial Artery	Anterior Tibial Vein Saphenous Vein	Failed
6	30	Female	Trauma	Forearm	11x5 cms	Radial Artery	Radial Vein Cephalic Vein	Survived
7	36	Male	Trauma	Forearm	7x5 cms	Radial Artery	Radial Vein Cephalic Vein	Survived
8	28	Female	Dermatofibrosarcoma Protuberance	Forearm	10x6 cms	Radial Artery	Radial Vein Cephalic Vein	Survived
9	33	Female	Post Burn Contracture	Neck	18x8 cms	Superior Thyroid Artery	Common Facial Vein Internal Jugular Vein	Survived
10	24	Male	Post Burn Contracture	First Web Space	12x6 cms	Radial Artery	Radial Vein	Survived
11	40	Male	Squamous Cell Carcinoma	Lip and Cheek	19x6 cms	Facial Artery	Internal Jugular Vein External Jugular Vein	Survived
12	54	Male	Squamous Cell Carcinoma	Buccal mucosa Lining	7x5 cms	Superior Thyroid Artery	Internal Jugular Vein External Jugular Vein	Survived
13	49	Male	Post Burn Contracture	First Web Space and Second Web Space and Palm	15x6 cms	Radial Artery	Cephalic Vein Superficial Volar Vein	Survived
14	40	Male	Squamous Cell Carcinoma	Buccal Mucosa Lining and Cover	14x5 cms	Superior Thyroid Artery	Internal Jugular Vein	Survived
15	51	Male	Squamous Cell Carcinoma	Buccal Mucosa Lining	6x4 cms	Superior Thyroid Artery	Internal Jugular Vein	Failed
16	40	Female	Squamous Cell Carcinoma	Upper lip and Commissure	11x6 cms	Superior Thyroid Artery	Internal Jugular Vein	Survived
17	68	Male	Squamous Cell Carcinoma	Tongue	12x8 cms	Superior Thyroid Artery	Common Facial Vein	Survived
18	58	Male	Squamous Cell Carcinoma	Tongue	9x5 cms	Superior Thyroid Artery	Internal Jugular Vein External Jugular Vein	Survived
19	38	Male	Squamous Cell Carcinoma	Tongue	14x5 cms	Superior Thyroid Artery	Internal Jugular Vein	Survived
20	51	Male	Squamous Cell Carcinoma	Tongue	13x6 cms	Superior Thyroid Artery	Internal Jugular Vein External Jugular Vein	Survived
21	39	Female	Squamous Cell Carcinoma	Tongue	12x5 cms	Superior Thyroid Artery	Internal Jugular Vein	Survived
22	84	Male	Squamous Cell Carcinoma	Tongue	14x6 cms	Superior Thyroid Artery	Internal Jugular Vein External Jugular Vein	Survived
23	87	Female	Squamous Cell Carcinoma	Tongue	13x5 cms	Superior Thyroid Artery,	Internal Jugular Vein External Jugular Vein	Survived

**Table 2:** Clinical characteristics, surgical details, and outcomes of patients who underwent pedicled lateral arm flap reconstruction (PLAP)

S.No	Age in years	Gender	Aetiology	Location of Defect	Flap Size	Outcome
1.	26	Female	Post Burn Contracture	Elbow Joint	9x5cms	Survived
2.	19	Female	Post Burn Contracture	Elbow Joint	9x4.5cms	Survived
3.	14	Female	Plate Exposure	Olecranon Process of Ulna	11x6cms	Survived
4.	16	Male	Traumatic Wound	Antecubital Fossa	9x4.5cms	Survived
5.	06	Female	Post Burn Contracture	Elbow Joint	5x3.5cms	Survived
6.	08	Male	Post Burn Contracture	Elbow Joint	5x3.5cms	Survived
7.	13	Male	Post Burn Contracture	Elbow Joint	8x5cms	Survived
8.	17	Male	Traumatic Contracture	Elbow Joint	8x6cms	Survived
9.	23	Male	Traumatic Wound	Antecubital Fossa	9x5cms	Survived
10.	26	Female	Post Burn Contracture	Elbow Joint	10x5cms	Survived

The average age of the patients in Group 1 was 43 years (14-87 years) while it was 17 years (6-26 years) in Group 2. In Group 1, 17 out of the 23 patients were males and six were females; Group 2 consisted of 5 males and five females.

In all, seven free flaps reconstruction were performed for the tongue, four for the reconstruction of the dorsum of the hand, three for the forearm, three for the buccal mucosa lining, two for combined defect lips/cheek and lip/commissure, one each for the lower limb, first web space, a combined defect involving the first web space, second webspace, and palm of the hand, and one for the neck. The etiology of the defect was found to be squamous cell carcinoma in 12 cases, trauma in six cases, post-burn contracture in four cases, and dermatofibrosarcoma protuberance in one case. The average free flap size was12x6 cm (range: 7x5 cm to 18x8 cm). In two patients, the extended lateral arm free flap (ELAF) was performed, which is a variation of the FLAF.

For Head and neck cases the superior thyroid artery was used as a recipient artery in 12 cases and facial artery in one case. For the hand, the radial artery was used in all nine cases, For the lower limb, the anterior tibial artery was used.

In 13 cases, two veins were anastomosed while in 10 cases, single vein anastomosis was performed. For head and neck, end-to-side anastomosis was performed commonly with the internal jugular vein. The external jugular vein was used as a second vein in a few cases. In the hand, the cephalic vein and venae comitantes were the recipient veins. In the lower limb, saphenous vein and venae comitantes were used as the recipient veins.

The minor complications included donor site wound dehiscence. The major complication was flap failure. Flap failure was encountered in three cases, two of them occurred in the early part of the series. These two cases had arterial insufficiency secondary to the anastomosis in the zone of injury. One flap failed due to infection on the fourth postoperative day in the later part of the series. All donor sites were closed primarily, but one patient encountered dehiscence secondary to necrosis at the wound edges because of closure under tension.

In Group 2, there were 10 patients. Six flaps were performed for the release of antecubital fossa post-burn contracture. Three were carried out for the traumatic wound of antecubital fossa that required flap coverage, and one was performed for plate exposure at the elbow after an olecranon fracture. The average pedicled flap size was 8x5 cm. All donor sites were closed primarily but the donor sites near the vascular supply had to be opened in three cases to relieve postoperative venous congestion. All flaps in Group 2 survived (Tables 1-2).

Case 1

**Figure 6:**
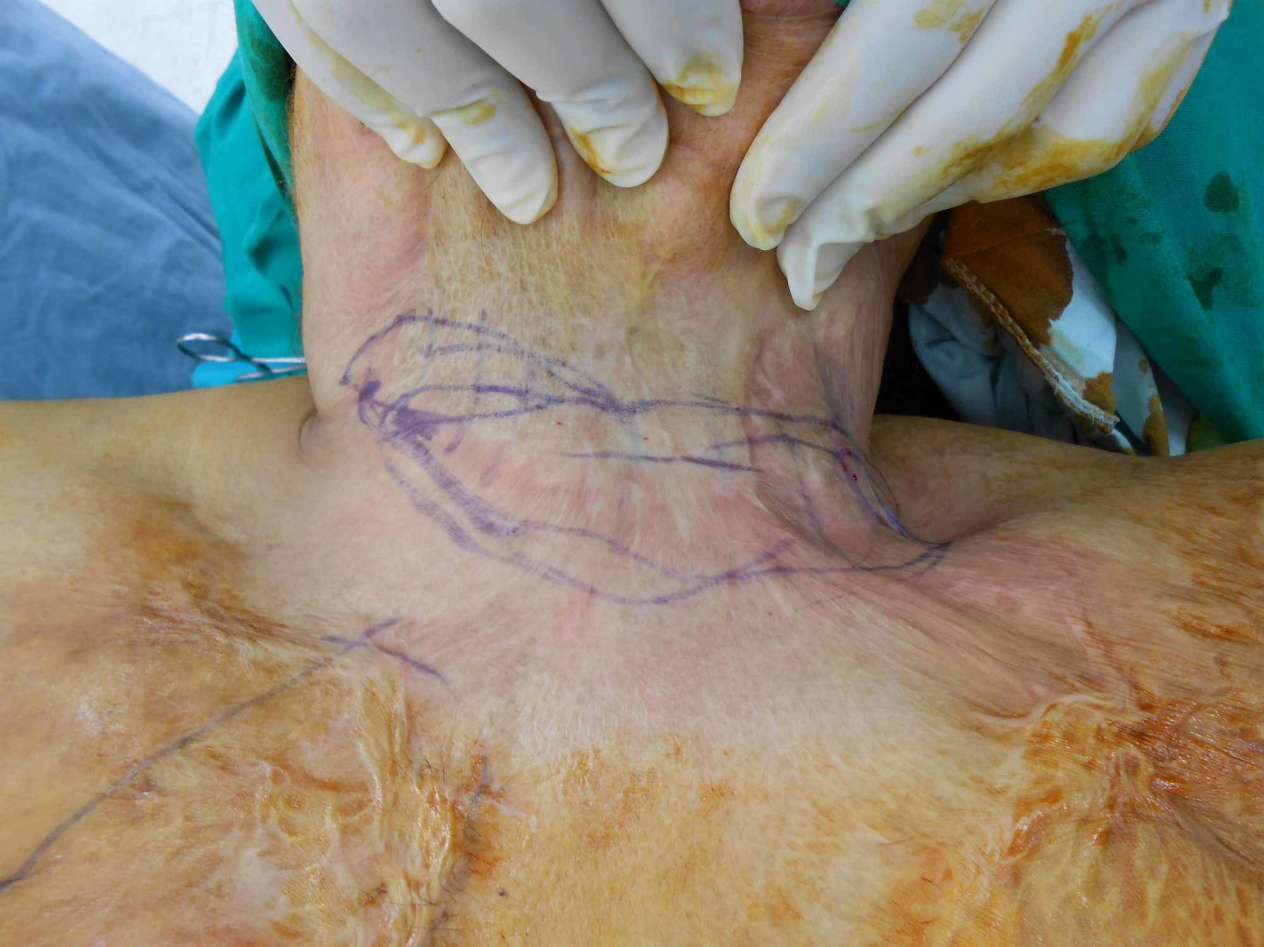
Preop presentation neck contracture

**Figure 7:**
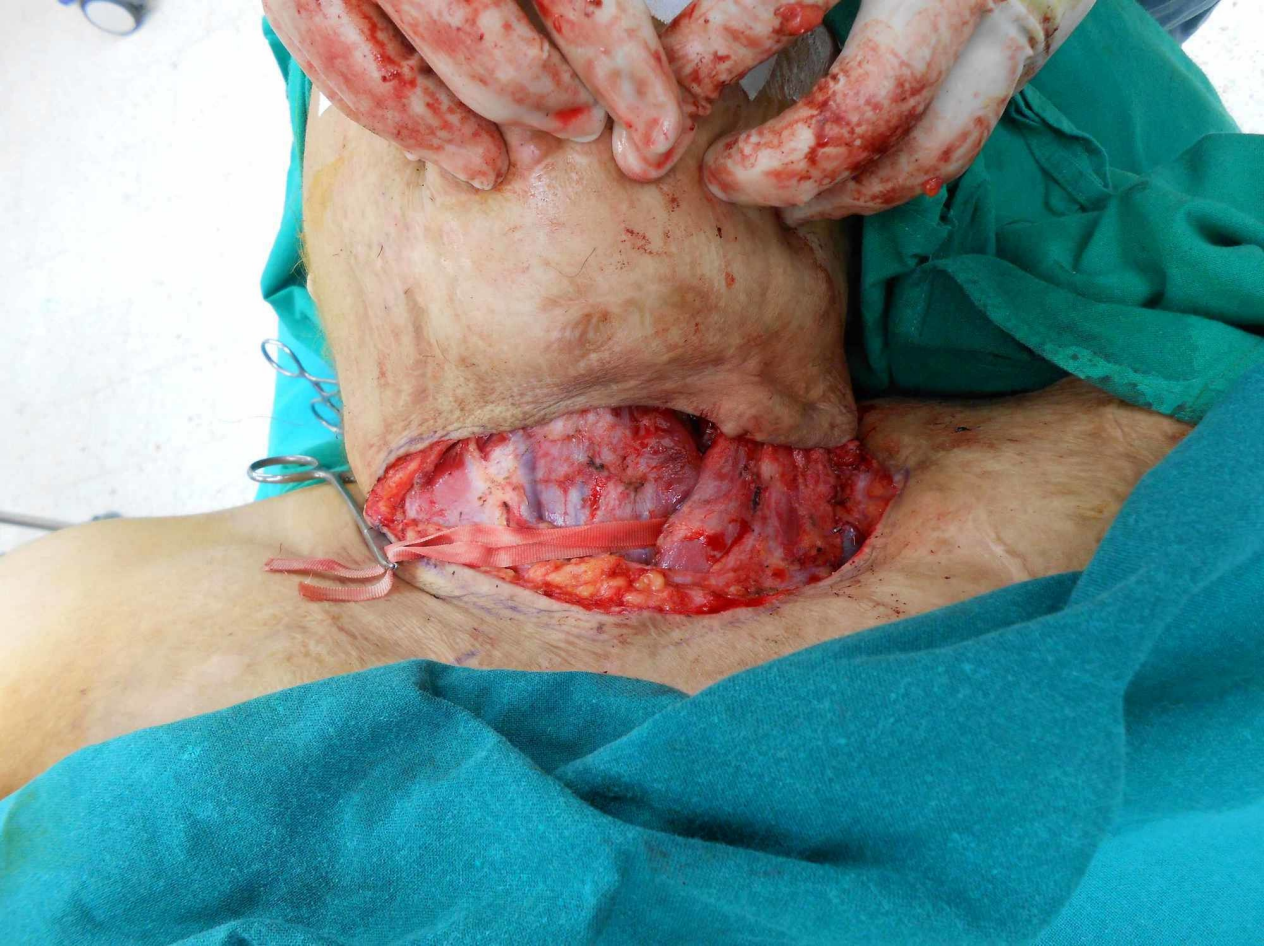
Intraop after release of the contracture

**Figure 8:**
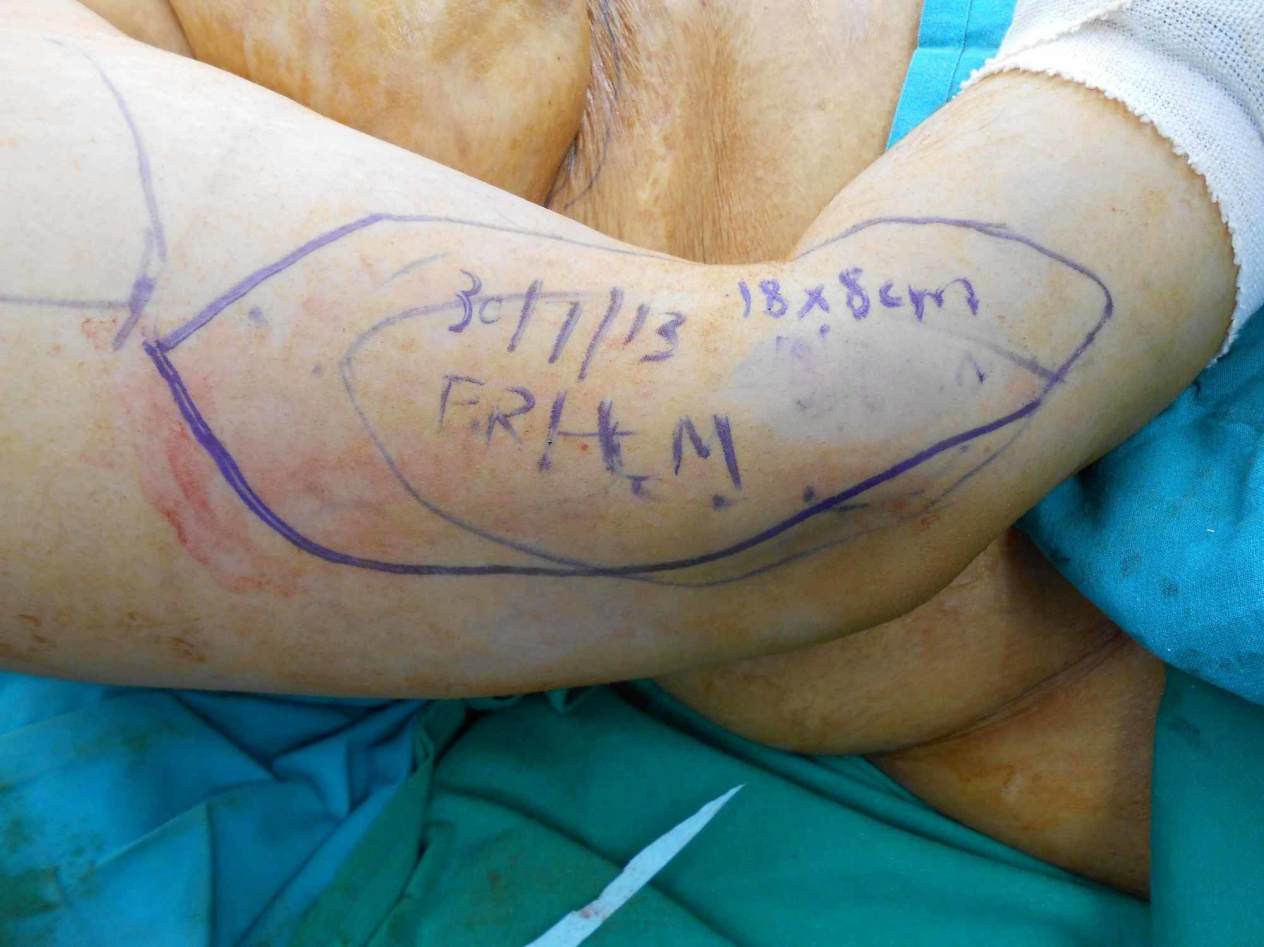
Flap marking

**Figure 9:**
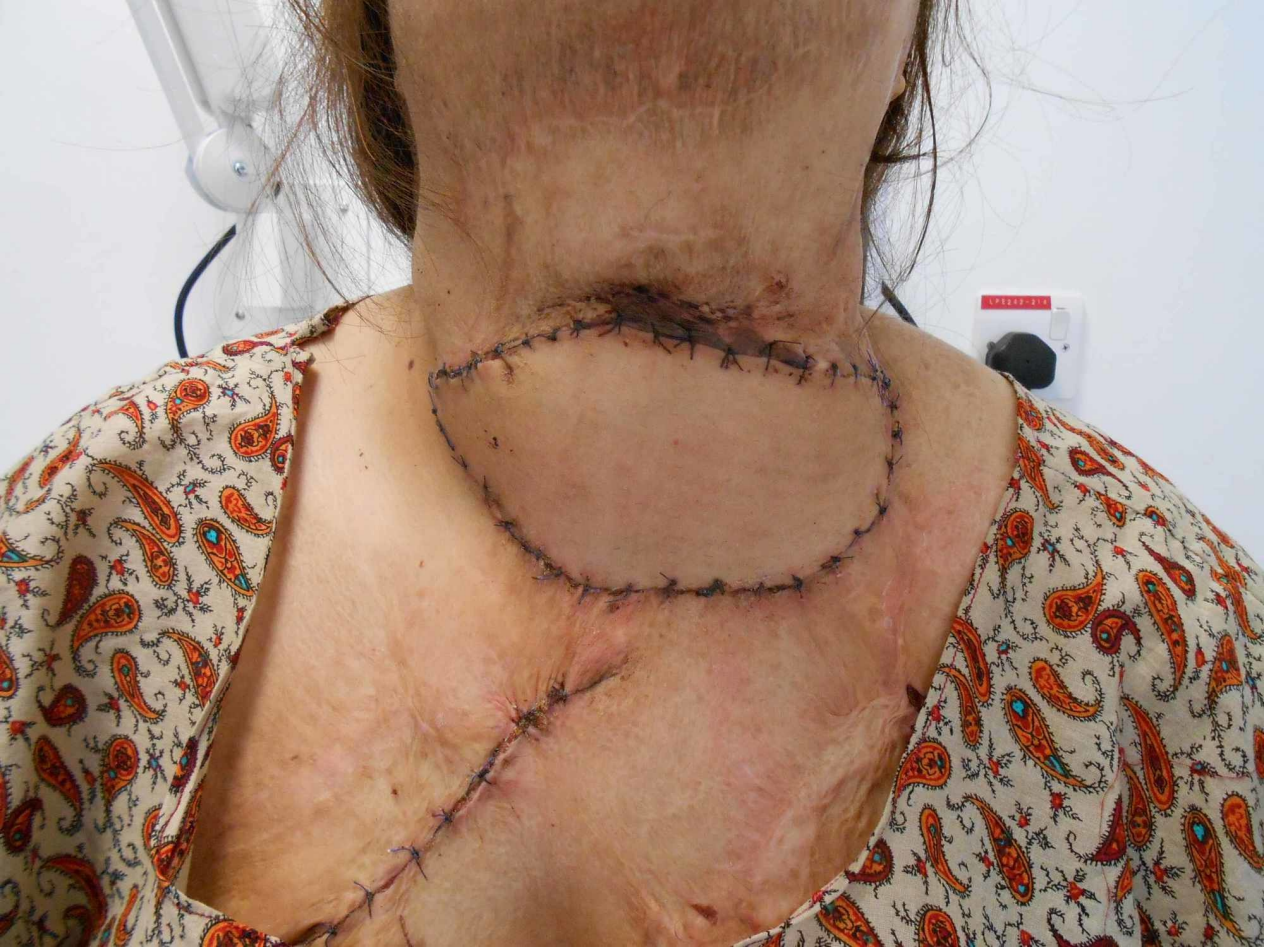
Follow-up two weeks

A 33-year-old female presented to us with post burn contracture for 19 years involving the neck, chest, and the bilateral axilla (Figure 6). The neck contracture involved upto 2/3rds of the anterior neck, with a pull on the left side of the lower lip. No extension was possible; however, she could flex her neck. The surgical procedure involved release of the neck contracture, followed by reconstruction with a free extended lateral arm flap, measuring 18x8 cms (Figure 7-9). The artery was anastomosed end-to-end using the superior thyroid artery, and both the venae comitantes were anastomosed end-to-end with a tributary of the internal jugular vein and end-to-side with the internal jugular vein. Contractures involving other areas were managed with ancillary procedures.

Case 2

**Figure 10:**
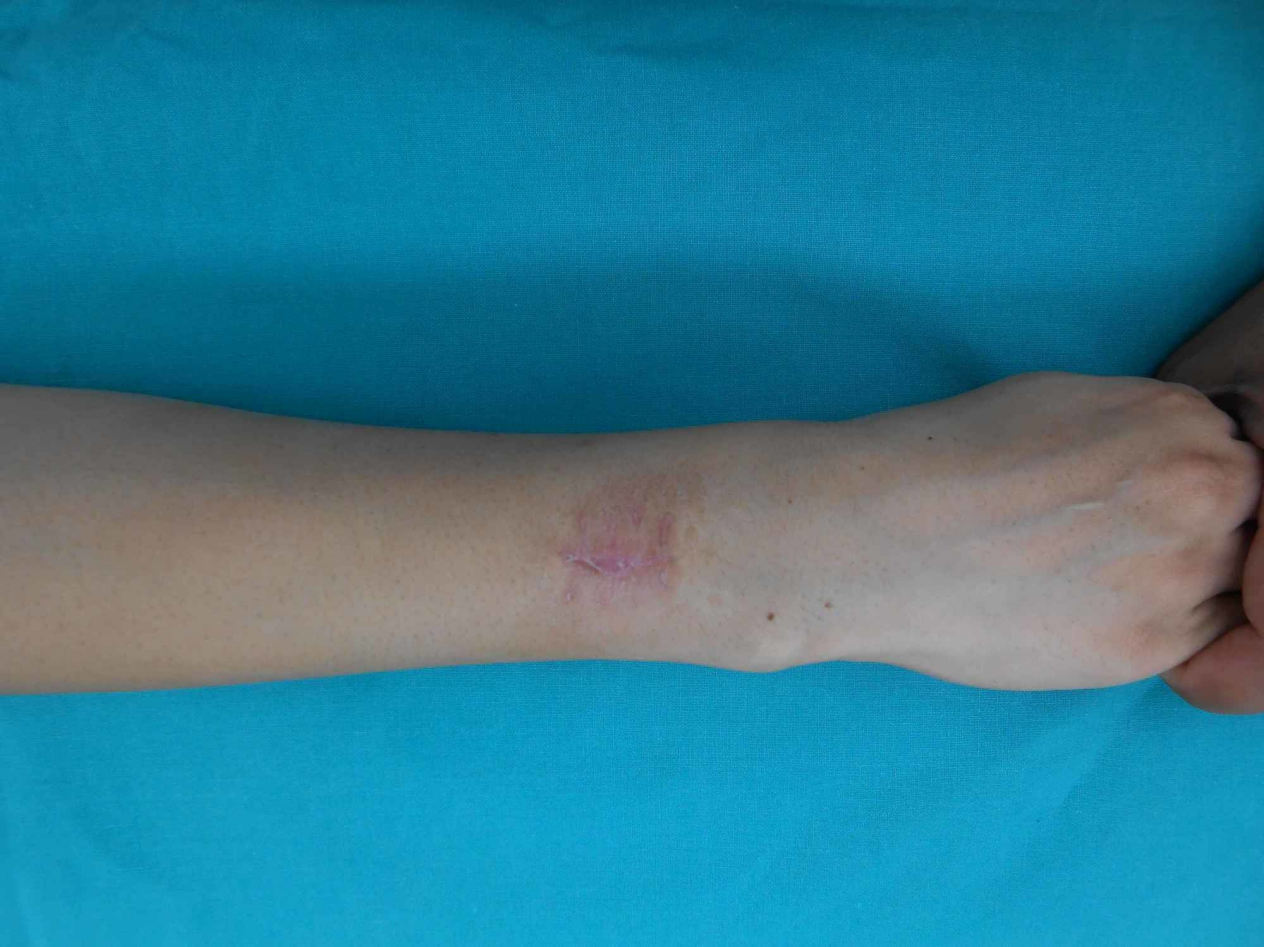
Preop scar forearm

**Figure 11:**
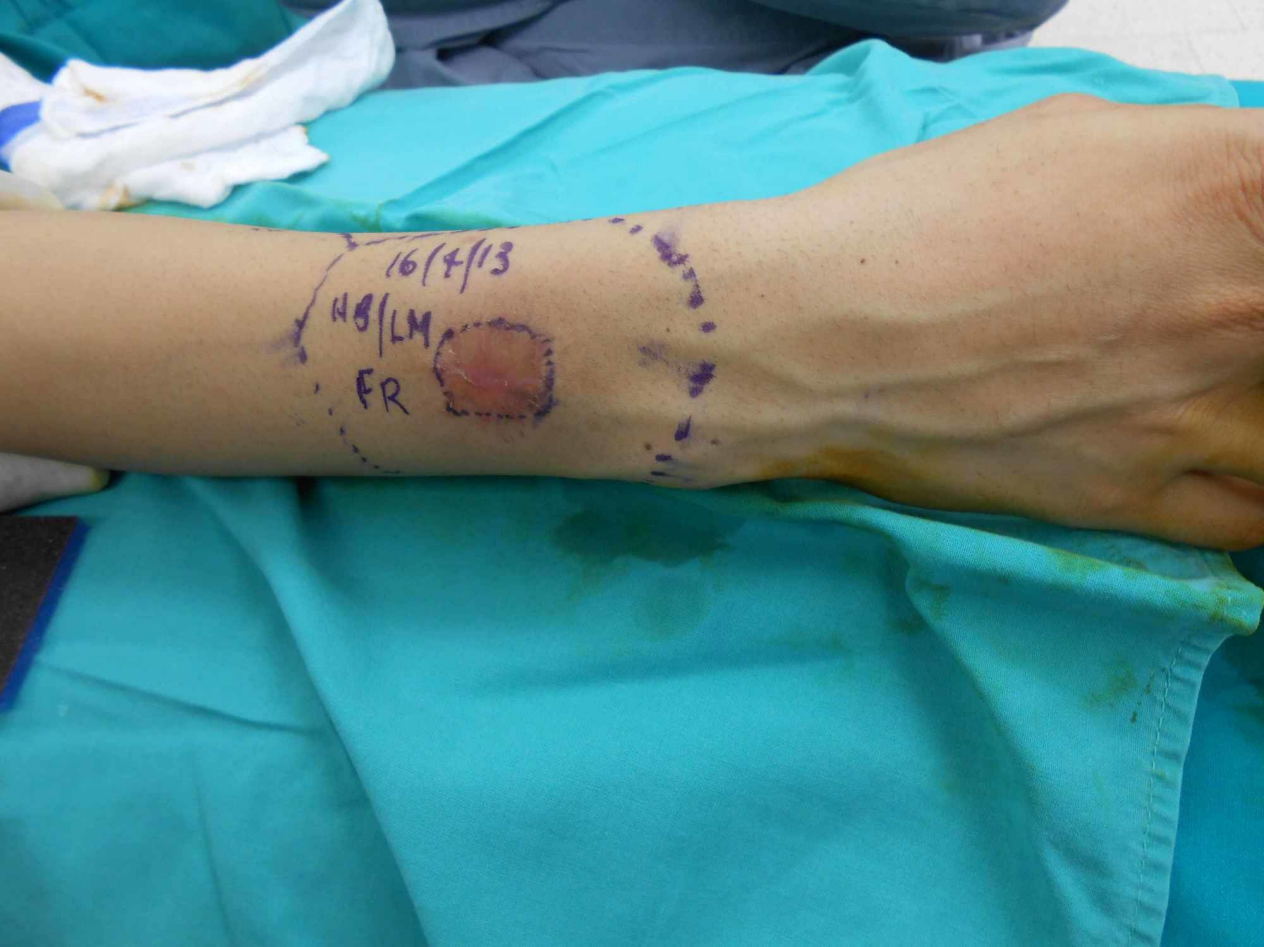
Markings for excision

**Figure 12:**
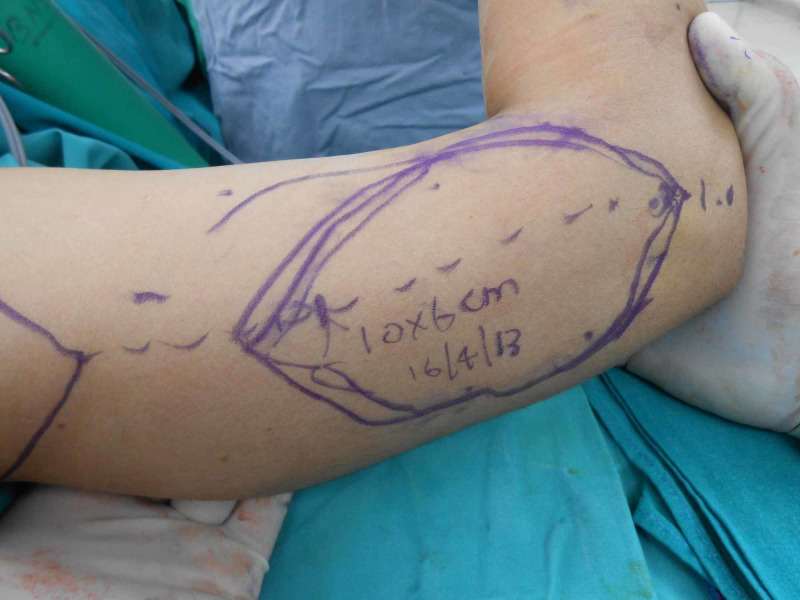
Flap marking

**Figure 13:**
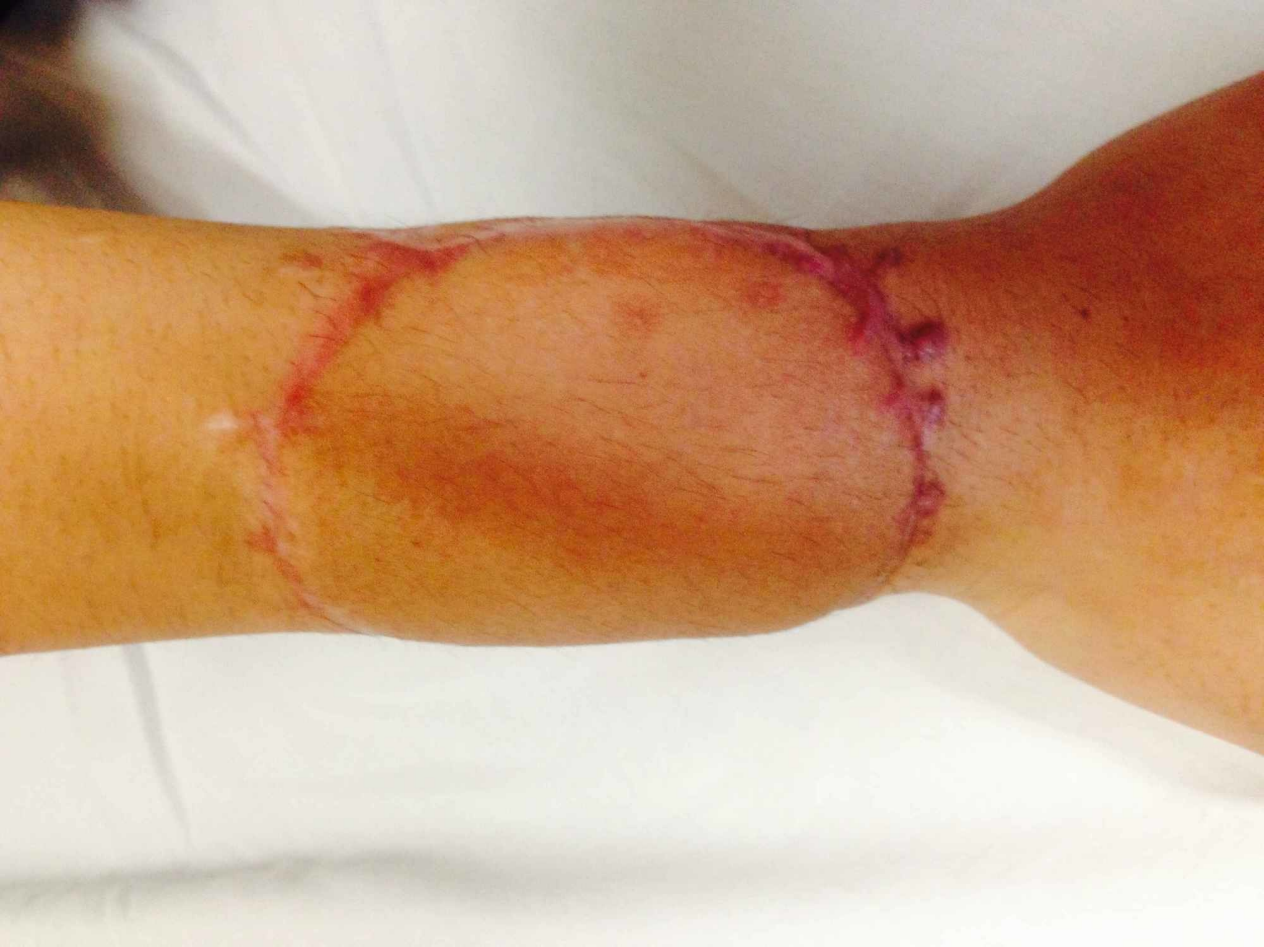
One and a half year postoperatively

A 28-year-old female presented to us with a scar on the dorsal aspect of the forearm (Figure 10). The patient had a lesion at the same site, which had been there for about a year. On excisional biopsy, the histopathology of the lesion revealed a dermatofibrosarcoma Ppotuberance, so she was planned for wide local excision of the scar and a free lateral arm flap (Figures 11-12). The defect arising upon the excision of the lesion was 10x6 cm, which was covered with a free arm lateral flap of the same size (Figure 13). Arterial anastomosis was carried out end-to-end with the radial artery; one vein was anastomosed end-to-end with the venae comitantes of the radial artery and the other with the cephalic vein.

## Discussion

The free lateral arm flap is a good option for the reconstruction of simple and complex defects of small to medium size, on different parts of the body. The flap has constant anatomy and a relatively longer vascular pedicle besides the great variety of tissue components that are included. For cases with both head and neck, team change of position during surgery is usually not necessary, and the operation time can be lowered by two teams working simultaneously. Secondary debulking procedures are not frequently required. For an aesthetically satisfactory result, the primary closure of the donor defect is worth aspiring for [8].

The rate of revision, complication, or flap loss is low; however, with a long learning curve. In our series, we lost three flaps. Two of these failures occurred in the first five flaps of the series, which points to the learning curve as compared to other commonly performed flaps. The long learning curve is because of its tedious dissection and small caliber of the vessels, which are usually 1.5 mm in diameter [9].

Since its initial description, there have been a number of modifications in the lateral arm flap. The ELAF can be harvested by designing the flap distal to the lateral epicondyle, along the axis, from the lateral epicondyle to the radial styloid [10]. This modification not only increases the length of the flap but also allows for increased length of the pedicle. In our series, there were two cases in which the ELAF was used. Other modifications include harvesting a cortex of the humerus, triceps muscle tendon, and the posterior cutaneous nerve of the forearm, along with the flap [11]. All these modifications lead to the reconstruction of a variety of defects with the lateral arm flap.

Another important variation of the lateral arm flap is the pedicled reverse lateral arm flap. A patient with a defect in the cubital fossa or olecranon area needs soft pliable tissue. Pedicle reverse lateral arm flaps provide stable soft tissue coverage, in conjunction with a consistent axial pedicle. With careful preoperative planning, this flap is a safe and reliable method for the reconstruction of complex elbow injuries, as long as the distal supply to the flap is intact. This flap was used in three patients with acute traumatic wounds on the elbow, in our series, without any complications [12].

One of the most important factors in choosing an appropriate flap for a particular defect is the donor site outcome.

In a retrospective survey, Graham et al. analyzed the donor site morbidity of 123 LAFs and found the appearance of the scar on the lateral arm to be the major reason for dissatisfaction [13]. This was especially the case when skin grafts were used to cover the donor site and when patients were females. The second major problem that was reported was lateral epicondylar pain.

Contrary to the findings of Graham et al., Depner et al. found scar visibility to be well-accepted by the patients in their study, with no differences with respect to gender. A possible reason for this discrepancy, according to them, could be the fact that they preferred to avoid skin grafting of the donor-site defect by limiting the flap width to 6 or 7 cm, thereby, allowing primary wound closure [14].

We achieved the primary closure of the donor site in all patients. However, three of the patients had a flap width greater than 6 cm because of large arm circumference so the donor site was closed primarily.

## Conclusions

We that the lateral arm flap can be used both as a pedicled and as a free flap. It has minimal donor site morbidity and is a useful addition to the armamentarium of a reconstructive surgeon.
